# Trends in gastrointestinal infections before and during non-pharmaceutical interventions in Korea in comparison with the United States

**DOI:** 10.4178/epih.e2022011

**Published:** 2022-01-03

**Authors:** Soyeoun Kim, Jinhyun Kim, Bo Youl Choi, Boyoung Park

**Affiliations:** 1 Department of Health Sciences, Hanyang University College of Medicine, Seoul, Korea; 2 Economics & Business Economics, University of Amsterdam, Amsterdam, The Netherlands; 3 Department of Preventive Medicine, Hanyang University College of Medicine, Seoul, Korea

**Keywords:** COVID-19, Gastrointestinal diseases, Personal protective equipment

## Abstract

**OBJECTIVES:**

This study examined how trends in the weekly frequencies of gastrointestinal infectious diseases changed before and during the coronavirus disease 2019 (COVID-19) pandemic in Korea, and compared them with the trends in the United States.

**METHODS:**

We compared the weekly frequencies of gastrointestinal infectious diseases (16 bacterial and 6 viral diseases) in Korea during weeks 5-52 before and after COVID-19. In addition, the weekly frequencies of 5 gastrointestinal infectious diseases in the United States (data from the Centers for Disease Control and Prevention) that overlapped with those in Korea were compared.

**RESULTS:**

The mean weekly number of total cases of gastrointestinal infectious diseases in Korea showed a significant decrease (from 522 before COVID-19 to 245 after COVID-19, p<0.01). Only bacterial gastrointestinal infectious diseases caused by *Campylobacter* increased significantly; other bacterial gastrointestinal infectious diseases showed either a decrease or no change. The incidence of all other viral diseases decreased. In the United States, the weekly numbers of *Salmonella*, *Campylobacter*, typhoid, shigellosis, and hepatitis A virus cases sharply decreased after the COVID-19 outbreak. The weekly case numbers of all viral diseases markedly decreased in both countries; however, bacterial gastrointestinal infectious diseases showed a different pattern.

**CONCLUSIONS:**

The incidence of gastrointestinal infectious diseases decreased after the COVID-19 outbreak. In contrast, *Campylobacter* infections showed an increasing trend in Korea, but a decreasing trend in the United States. Further studies are needed to elucidate the different trends in bacterial and viral infectious diseases before and after non-pharmaceutical interventions and between different countries.

## GRAPHICAL ABSTRACT

[Fig f4-epih-44-e2022011]

## INTRODUCTION

On March 11, 2020, the World Health Organization (WHO) declared coronavirus disease 2019 (COVID-19) as a pandemic. After the WHO declaration, non-pharmacological interventions (NPIs) against COVID-19 were implemented in several countries [1]. NPIs such as movement restrictions and isolation measures began in March 2020 worldwide [2], and Korea also started implementing nationwide NPIs in March 2020 [3,4]. As a result of NPIs, the weekly positivity rates of viral respiratory infections significantly decreased in 2020 compared with those in the same weeks in 2010-2019 [5]. Interventions, including personal hygiene and social distancing measures, might affect infectious diseases other than respiratory infectious diseases. However, whether NPIs could influence the development of other infectious diseases is unknown owing to the limited number of studies. Therefore, we examined how trends in the weekly frequencies of gastrointestinal infectious diseases changed before and during the COVID-19 pandemic in Korea, considering trends in COVID-19 incidence, and compared them with the trends in the United States.

## MATERIALS AND METHODS

We analyzed the weekly number of cases of gastrointestinal infectious diseases from the Infectious Disease Surveillance System established by the Korea Disease Control and Prevention Agency (KDCA; formerly the Korea Centers for Disease Control and Prevention). Physicians reported cases to this surveillance system based on the Infectious Disease Control and Prevention Act [6]. After collecting data from the local health authorities, these data were transferred to the KDCA. A report of national statistics was then published using confirmed data from the KDCA [7]. According to reporting guidelines, reported infectious diseases are classified into 3 groups; pathogen identified, suspected infection, or a pathogen carrier [8]. The infectious disease surveillance system in Korea is composed of a mandatory surveillance system and a sentinel surveillance system [6,9]. The mandatory surveillance system requires obligatory reporting from all health centers immediately or within 24 hours of identifying 64 types of infectious diseases. The sentinel surveillance system receives weekly reports on 23 types of infectious diseases from designated health institutions. In this study, we considered gastrointestinal infectious diseases, including cholera, shigellosis, typhoid, paratyphoid, enterohemorrhagic *Escherichia coli* (EHEC), and hepatitis A virus, which are collected through the mandatory surveillance system: *Salmonella*, enteroinvasive *E. coli* (EIEC), *Yersinia enterocolitica*, *Clostridium perfringens*, *Vibrio parahaemolyticus*, enteropathogenic *E. coli* (EPEC), *Bacillus cereus*, *Campylobacter*, enterotoxigenic *E. coli* (ETEC), *Listeria monocytogenes*, *Staphylococcus aureus*, group A rotavirus, astrovirus, adenovirus, norovirus, and sapovirus infections, which are collected through the sentinel surveillance system [9]. For a cross-country comparison with the United States, we used weekly reports from the Nationally Notifiable Diseases Surveillance System (NNDSS) administered by the United States Centers for Disease Control and Prevention (CDC) [10], to which infectious and contagious diseases are immediately reported by healthcare providers [11,12]. The NNDSS is collected from all 50 states and the District of Columbia and monitors about 120 diseases classified by the main transmission: sexually transmitted, foodborne or waterborne, vector borne, injection drug use–associated, and respiratory [13]. As a result, the types of infectious agents reported in the KDCA in Korea and the CDC in the United States differed. Thus, we selected infectious diseases from the CDC that overlapped with those in the KDCA for comparison: salmonellosis, *Campylobacter* infection, typhoid, shigellosis, and hepatitis A virus infection. For the period before COVID-19, the mandatory surveillance system used the mean weekly number of cases from 2015 to 2019, which was calculated as the sum of all weekly case numbers divided by 5 (the number of years). The sentinel surveillance system used data from July 30, 2017 to 2019, considering the expansion of medical centers for sentinel surveillance in July 2017. For the period after COVID-19, we used the weekly number of cases of each type of gastrointestinal infectious disease from weeks 5-52 of 2020. We then compared the mean weekly case number for the same weeks before and after COVID-19 in the mandatory surveillance system and the sentinel surveillance system using the paired t-test.

We excluded 1-4 weeks from each year to reflect the incidence of COVID-19 because the first COVID-19 patient was identified in Korea on January 20, 2020. Data on the number of weekly COVID-19 cases from the CDC in the United States [14] and from the KDCA in Korea [3] were used. For visualization, we plotted the trends in the weekly number of new COVID-19 cases together with the mean weekly number of each gastrointestinal infectious disease for 2 periods (before and after COVID-19). All statistical analyses were conducted using Microsoft Excel (version 2016; Microsoft, Redmond, WA, USA).

### Ethics statement

This study used from official websites which are opened to the public. In accordance with Article 13 of the Enforcement Ordinance, this study is subject to institutional review board exception.

## RESULTS

Table 1 shows the mean weekly number of cases of gastrointestinal infectious diseases during weeks 5-52 before COVID-19 compared with that after COVID-19 in Korea and the United States. In Korea, the weekly case number of total gastrointestinal infectious diseases showed a significant decrease (from 522 before COVID-19 to 245 after COVID-19, p< 0.01). Statistically significant decreases were observed for all types of viral gastrointestinal infectious diseases (range of decrease: -55.2 to -5.8, p< 0.01). The decreases for total bacterial gastrointestinal infectious diseases were not statistically significant (p= 0.12). In contrast, the number of total viral gastrointestinal infectious diseases markedly decreased after COVID-19 compared with the period before COVID-19. Meanwhile, in the United States, the number of cases significantly decreased for both total bacterial gastrointestinal diseases and total viral gastrointestinal diseases. In particular, in Korea, statistically significant decreases were observed for *Salmonella*, *V. parahaemolyticus*, ETEC, EIEC, *S. aureus*, *B. cereus*, typhoid, and shigellosis. A statistically significant increase was observed for *Campylobacter* infections. There were no statistically significant differences in *C. perfringens*, *Y. enterocolitica*, *Listeria monocytogenes*, EHEC, cholera, paratyphoid, and EPEC infections. In the United States, significant decreases were found for salmonellosis (-151.8), *Campylobacter* infection (-163.9), typhoid (-1.5), shigellosis (-71.9), and hepatitis A virus infection (-15.5) after COVID-19 compared before COVID-19 (p< 0.01 for all).

**Figure 1.**
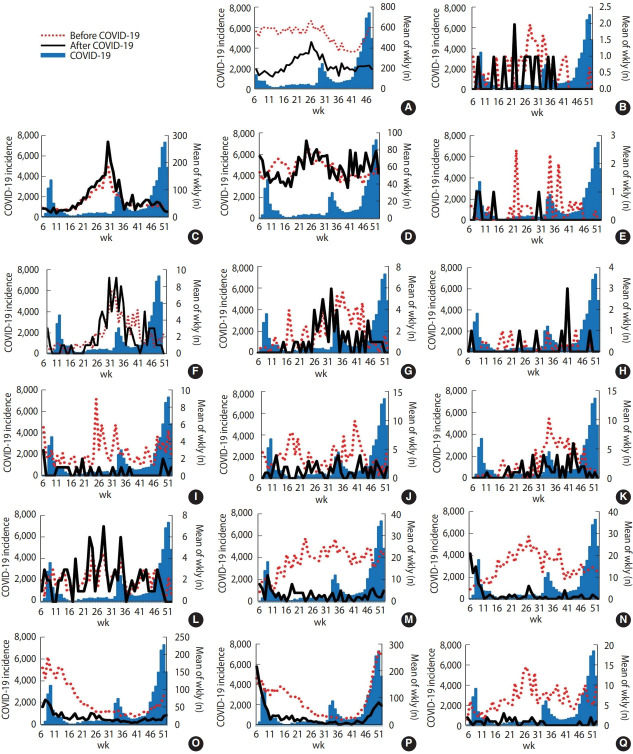
Mean weekly numbers of gastrointestinal infections identified by sentinel surveillance in Korea before coronavirus disease 2019 (COVID-19) compared with after COVID-19. The red dotted lines indicate the mean weekly incidence in Korea before COVID-19. The black lines show the weekly incidence after COVID-19. The blue zones indicate the COVID-19 incidence in Korea. (A) Total gastrointestinal infectious diseases. (B) *Bacillus cereus*. (C) *Campylobacter*. (D) *Clostridium perfringens*. (E) Enteroinvasive *Escherichia coli*. (F) Enteropathogenic *E. coli*. (G) Enterotoxigenic *E. coli*. (H) *Listeria monocytogenes*. (I) *Salmonella*. (J) *Staphylococcus aureus*. (K) *Vibrio parahaemolyticus*. (L) *Yersinia enterocolitica*. (M) Adenovirus. (N) Astrovirus. (O) Group A rotaviruses. (P) Norovirus. (Q) Sapovirus.

**Figure 2.**
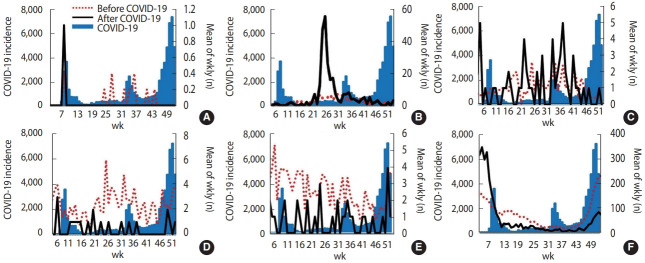
Mean weekly numbers of gastrointestinal infections by mandatory surveillance in Korea before coronavirus disease 2019 (COVID-19) compared with after COVID-19. The red dotted lines indicate the mean weekly incidence in Korea before COVID-19. The black lines show the weekly incidence after COVID-19. The blue zones indicate the COVID-19 incidence in Korea. (A) Cholera. (B) Enterohemorrhagic *Escherichia coli*. (C) Paratyphoid. (D) Shigellosis. (E) Typhoid. (F) Hepatitis A.

Figures 1 and 2 present the epidemic curves of each type of gastrointestinal infectious disease determined by sentinel surveillance and mandatory surveillance during weeks 5–52 before and after COVID-19 in Korea with the weekly number of new COVID-19 cases. After the start of the COVID-19 outbreak, followed by the establishment of NPIs in Korea, the frequency of the 6 types of viral diseases decreased, especially group A rotavirus, astrovirus, adenovirus, norovirus, and sapovirus infections. In particular, the frequency of astrovirus and sapovirus infections reached nearly 0 from week 41. The epidemic patterns of bacterial diseases did not show a dramatic decrease after COVID-19.

**Figure 3.**
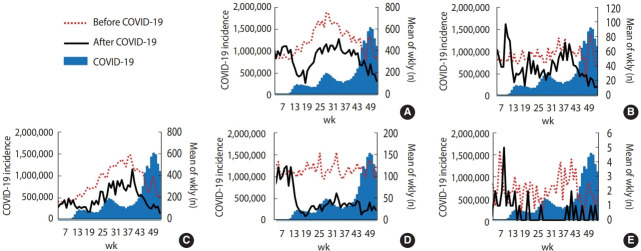
Mean weekly numbers of gastrointestinal infections in the United States during 2015-2019 compared with those in 2020. The red dotted lines indicate the mean weekly incidence in the United States before coronavirus disease 2019 (COVID-19). The black lines show the weekly incidence after COVID-19. The blue zones indicate the COVID-19 incidence in the United States. (A) *Campylobacter*. (B) Hepatitis A. (C) *Salmonella*. (D) Shigellosis. (E) Typhoid.

Figure 3 shows that the weekly case numbers of *Salmonella* infection, *Campylobacter* infection, typhoid, shigellosis, and hepatitis A virus infection sharply decreased after the COVID-19 outbreak in the United States. The decrease in the number of shigellosis cases was most pronounced, from 124 on week 9 to 9 on week 18.

## DISCUSSION

With respect to the 5 gastrointestinal infections that were collected in both countries, not only the weekly case numbers of each bacterial and viral disease, but also the total case number of bacterial and viral gastrointestinal diseases markedly decreased in the United States. However, Korea showed a different pattern. Specifically, the total number of viral gastrointestinal diseases decreased significantly, while the total number of bacterial gastrointestinal diseases did not significantly decrease. In addition, each bacterial gastrointestinal disease had distinct trends.

Infectious diseases are expected to decrease after the COVID-19 pandemic due to worldwide NPIs. In Korea, the total number of viral gastrointestinal infections showed a considerable decrease, but the decreases in the total bacterial gastrointestinal infections did not reach a statistically significant level. Unlike the viral gastrointestinal infectious diseases, there were fluctuations in the trends of bacterial gastrointestinal infections (downward, stable, and then upward) in 2020 compared with previous years. Significant decreases were observed for *Salmonella*, *V. parahaemolyticus*, ETEC, EIEC, *S. aureus*, *B. cereus*, typhoid, and shigellosis. However, only the increase in *Campylobacter* infections was statistically significant. NPIs, such as hand hygiene and wearing a mask, can reduce the spread of viral diseases [15], and these viral gastrointestinal infectious diseases also showed a significant decrease like that observed for respiratory viruses, suggesting the existence of a foodborne or waterborne infection route for gastrointestinal viruses [16,17]. However, *C. perfringens* and EHEC did not show significant increases. This finding was probably due to an unstable small number. In the case of EHEC, the outbreak in 2019 may have affected the increase in EHEC [18]. Bacterial gastrointestinal infections were difficult to identify both before and after the COVID-19 outbreak. Bacterial pathogens propagate through rotten food or water, especially during the summer [19,20].

Since the types of gastrointestinal infections collected in Korea and the United States differed, the possibility of a direct comparison would be limited; however, there are several potential reasons for the observed divergence. First, the United States issued stay-athome orders between March and April 2020. In Korea, personal hygiene measures, such as mask-wearing, were used. Second, compared to the United States, Korea implemented a less strict social distancing policy, and therefore, accessibility to medical care may have been lower in the United States than in Korea [7]. Consequently, people who had gastrointestinal infectious diseases with less severe symptoms might have had a limited ability to access medical centers during the COVID-19 pandemic than during the period before it, which could have had a greater impact in the United States than in Korea. Thus, despite the possibility of underreporting followed by underestimation of actual infection numbers in both countries, this would be more impactful in the United States [21]. Despite these potential reasons, *Campylobacter* infections showed clearly different patterns between Korea and the United States. Gastrointestinal infectious diseases have a wide range of infection pathways. The agents of *Campylobacter* infections are animals, including dogs, cats, pigs, sheep, rodents, birds, and poultry [9]. According to a Korean study, retail raw chickens were confirmed in about 68% of cases for *Campylobacter*. After the COVID-19 outbreak, the annual consumption of chicken per adult increased by 1.2 kg compared to COVID-19 [22]. Thus, *Campylobacter* infections are a zoonotic contagious disease that can infect humans through food based on these assumptions. Additionally, in Korea, cluster outbreaks in restaurants accounted for the highest number of cases before COVID-19; however, after COVID-19, the most common place of cluster outbreaks shifted to pre-primary schools [9]. In Korea, kindergartens and pre-primary schools have different purposes. Kindergartens focus on education, and pre-primary schools focus on childcare. Therefore, this could explain the poor control of *Campylobacter* in the context of COVID-19. Our data, including the mandatory surveillance and sentinel surveillance, and, in the case of sentinel surveillance, the number of hospitals (including public hospitals) collecting data, increased from July 30, 2017 [23]. Thus, we compared the periods before and after COVID-19 separately for each surveillance system.

In conclusion, while the incidence of viral gastrointestinal diseases, including hepatitis A, prominently decreased after COVID-19, bacterial diseases showed different trends in Korea and a decreasing trend in the United States. Further studies are needed to elucidate the different trends in bacterial and viral infectious diseases before and after NPIs and between different countries, as well as considering the characteristics of each pathogen.

## Figures and Tables

**Figure f4-epih-44-e2022011:**
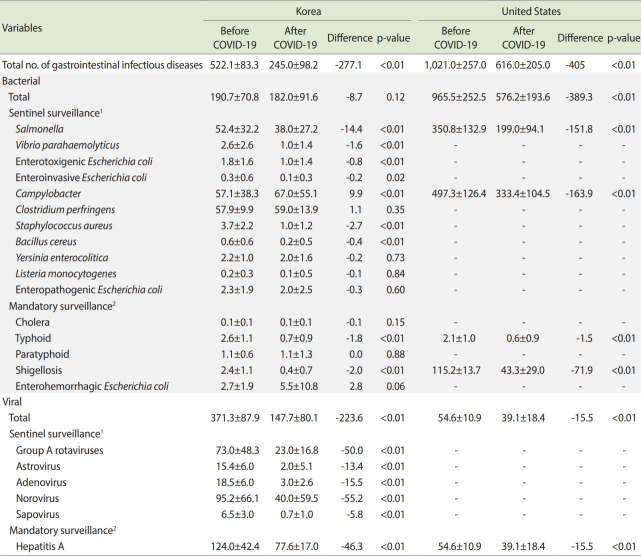


**Table 1.** Mean weekly numbers of cases gastrointestinal infectious diseases during weeks 5-52 before COVID-19 compared with the case numbers after COVID-19 in Korea and the United States

Variables			Korea				United States			
			Before COVID-19	After COVID-19	Difference	p-value	Before COVID-19	After COVID-19	Difference	p-value
Total no. of gastrointestinal infectious diseases			522.1±83.3	245.0±98.2	-277.1	<0.01	1,021.0±257.0	616.0±205.0	-405	<0.01
Bacterial										
	Total		190.7±70.8	182.0±91.6	-8.7	0.12	965.5±252.5	576.2±193.6	-389.3	<0.01
	Sentinel surveillance^1^									
		*Salmonella*	52.4±32.2	38.0±27.2	-14.4	<0.01	350.8±132.9	199.0±94.1	-151.8	<0.01
		*Vibrio parahaemolyticus*	2.6±2.6	1.0±1.4	-1.6	<0.01	-	-	-	-
		Enterotoxigenic *Escherichia coli*	1.8±1.6	1.0±1.4	-0.8	<0.01	-	-	-	-
		Enteroinvasive *Escherichia coli*	0.3±0.6	0.1±0.3	-0.2	0.02	-	-	-	-
		*Campylobacter*	57.1±38.3	67.0±55.1	9.9	<0.01	497.3±126.4	333.4±104.5	-163.9	<0.01
		*Clostridium perfringens*	57.9±9.9	59.0±13.9	1.1	0.35	-	-	-	-
		*Staphylococcus aureus*	3.7±2.2	1.0±1.2	-2.7	<0.01	-	-	-	-
		*Bacillus cereus*	0.6±0.6	0.2±0.5	-0.4	<0.01	-	-	-	-
		*Yersinia enterocolitica*	2.2±1.0	2.0±1.6	-0.2	0.73	-	-	-	-
		*Listeria monocytogenes*	0.2±0.3	0.1±0.5	-0.1	0.84	-	-	-	-
		Enteropathogenic *Escherichia coli*	2.3±1.9	2.0±2.5	-0.3	0.60	-	-	-	-
	Mandatory surveillance^2^									
		Cholera	0.1±0.1	0.1±0.1	-0.1	0.15	-	-	-	-
		Typhoid	2.6±1.1	0.7±0.9	-1.8	<0.01	2.1±1.0	0.6±0.9	-1.5	<0.01
		Paratyphoid	1.1±0.6	1.1±1.3	0.0	0.88	-	-	-	-
		Shigellosis	2.4±1.1	0.4±0.7	-2.0	<0.01	115.2±13.7	43.3±29.0	-71.9	<0.01
		Enterohemorrhagic *Escherichia coli*	2.7±1.9	5.5±10.8	2.8	0.06	-	-	-	-
Viral										
	Total		371.3±87.9	147.7±80.1	-223.6	<0.01	54.6±10.9	39.1±18.4	-15.5	<0.01
	Sentinel surveillance^1^									
		Group A rotaviruses	73.0±48.3	23.0±16.8	-50.0	<0.01	-	-	-	-
		Astrovirus	15.4±6.0	2.0±5.1	-13.4	<0.01	-	-	-	-
		Adenovirus	18.5±6.0	3.0±2.6	-15.5	<0.01	-	-	-	-
		Norovirus	95.2±66.1	40.0±59.5	-55.2	<0.01	-	-	-	-
		Sapovirus	6.5±3.0	0.7±1.0	-5.8	<0.01	-	-	-	-
	Mandatory surveillance^2^									
		Hepatitis A	124.0±42.4	77.6±17.0	-46.3	<0.01	54.6±10.9	39.1±18.4	-15.5	<0.01

Values are presented as mean weekly number±standard deviation.

1 The weekly number of each type of gastrointestinal infectious disease in weeks 5-52 of 2020 with the mean of the same weeks during July 30, 2017-2019 in Korea or during 2015-2019 in United States.

2 The weekly number of each type of gastrointestinal infectious disease in weeks 5-52 of 2020 with the mean of the same weeks during 2015-2019 in Korea and United States.
