# Erratum: Trends in gastrointestinal infections before and during non-pharmaceutical interventions in Korea in comparison with the United States

**DOI:** 10.4178/epih.e2022011.E

**Published:** 2022-12-31

**Authors:** Soyeoun Kim, Jinhyun Kim, Bo Youl Choi, Boyoung Park

**Affiliations:** 1 Department of Health Sciences, Hanyang University College of Medicine, Seoul, Korea; 2 Economics & Business Economics, University of Amsterdam, Amsterdam, The Netherlands; 3 Department of Preventive Medicine, Hanyang University College of Medicine, Seoul, Korea

To the Editor:

The authors regret that there was error in the text. This notice corrects the grant number in funding information.

Content of correction:

Present corrected the text in the one grant number of funding information.

This research was supported by the Government-wide R&D Fund Project for Infectious Disease Research (GFID), Republic of Korea (grant No. HG18C00880).

This research was supported by the Government-wide R&D Fund Project for Infectious Disease Research (GFID), Republic of Korea (grant No. HG18C0088). (Revised)

DOI of original article https://doi.org/10.4178/epih.e2022011

